# POTAGE: A Visualisation Tool for Speeding up Gene Discovery in Wheat

**DOI:** 10.1038/s41598-017-14591-7

**Published:** 2017-10-30

**Authors:** Radosław Suchecki, Nathan S. Watson-Haigh, Ute Baumann

**Affiliations:** University of Adelaide, School of Agriculture, Food and Wine, PMB 1, Glen Osmond, SA 5064 Australia

## Abstract

POPSEQ Ordered *Triticum aestivum* Gene Expression (POTAGE) is a web application which accelerates the process of identifying candidate genes for quantitative trait loci (QTL) in hexaploid wheat. This is achieved by leveraging several of the most commonly used data sets in wheat research. These include the Chromosome Survey Sequences, their order along the chromosomes determined by the population sequencing (POPSEQ) approach, the gene predictions and RNA-Seq expression data. POTAGE aggregates those data sets and provides an intuitive interface for biologists to explore the expression of the predicted genes and their functional annotation in a chromosomal context. The interface accelerates some of the laborious and repetitive tasks commonly undertaken in the process of identifying and prioritising genes which may underlie QTL. We illustrate the utility of POTAGE by showing how a short-list of candidate genes can quickly be identified for a QTL linked to pre-harvest sprouting - a major cause of quality and yield loss in wheat production. The candidate genes identified using POTAGE included *TaMKK3*, which was recently reported as a causal gene for seed dormancy in wheat, and a mutation in its barley ortholog has been shown to reduce pre-harvest sprouting. POTAGE is available at http://crobiad.agwine.adelaide.edu.au/potage.

## Introduction

Common wheat (*Triticum aestivum*) is a major food crop providing much of the calorie and protein requirements of the world’s population, and is also a major traded commodity. Wheat research is hampered by large (16 Gbp), hexaploid and repetitive nature of its genome, more than 80% of which consists of transposable elements^1–3^. However, more progress has been made in recent years, largely due to a number of data sets generated by the International Wheat Genome Sequencing Consortium. The frequently used Chromosome Survey Sequences (CSS)^4^ are shotgun sequence assemblies of flow sorted chromosome arms (except for chromosome 3B which is assembled as a whole). The utility of the CSS was further enhanced by POPSEQ, a method which uses genetic segregation in a bi-parental population to order contigs along a chromosome^5,6^. Other key data sets include the high confidence (HC) predictions of genes within the CSS contigs as well as a bread wheat tissue series RNA-Seq data set (http://urgi.versailles.inra.fr/files/RNASeqWheat/)^2^. All the sequencing data has been generated from Chinese Spring, while the POPSEQ map is based on 90 doubled haploid individuals of the synthetic W7984 × Opata M85 population^7^. One way to accelerate progress in wheat research is to facilitate the identification of genes under agronomically relevant quantitative trait loci (QTL). This is often a tedious process despite the availability of the aforementioned data sets.

In addition to the available genomic resources, two web tools have been released in the recent years that allow users to explore wheat transcriptomic data and as such can contribute valuable information towards the prioritisation of candidate genes. The first of these tools, WheatExp (https://wheat.pw.usda.gov/WheatExp/)^8^ provides access to expression values from six data sets and is homoeolog-aware in the sense that it displays expression values for homeologous genes side-by-side wherever the homoeology relationship has been established. In the second tool, expression values from 16 wheat data sets can be visualised using the expVip system (http://www.wheat-expression.com/)^9^. This tool uses the HC gene predictions from the CSS as well as the gene predictions made on the recently released TGACv1 whole genome sequencing assembly of hexaploid wheat^3^. Neither of these tools, although useful in their own right, links the predicted genes to chromosomal regions. In POTAGE, the expression information for a given gene is placed within the context of the its chromosomal position, making POTAGE the first tool for hexaploid wheat which enables access to expression data in a chromosomal context. POTAGE is designed with consideration for the intrinsic limitations of the underlying data sets, which we briefly outline before presenting the features of our tool. We conclude by investigating a QTL for pre-harvest sprouting, where POTAGE enabled us to rapidly identify a promising set of candidate genes.

## The Tool

### Data integration

The data sets leveraged by POTAGE have some limitations which had to be considered in the context of their integration. The CSS data are highly fragmented with hundreds of thousands of contigs per chromosome which is reflected in the commonly used assembly contiguity measure N50, which for most chromosomes falls within 2–3 kb range. However, an advantage of these data is that the chromosome (and in most cases the chromosome arm) to which a given contig belongs is known with a high degree of confidence. The utility of CSS is further enhanced by the POPSEQ map. Even though it only assigns 20% of the CSS assembly (bp) to genetic position bins along the chromosomes, these assignments cover 60% of HC gene predictions.

POTAGE utilizes the HC gene predictions located within CSS contigs as well as the contigs’ location in the POPSEQ map. Gene predictions are linked with the available expressions values, which can come from various sources. The expression values loaded in the public instance of POTAGE were computed as we have described previously^10^. POTAGE is gene-centric, which means that most information is linked primarily to the HC identifiers. About half of the gene predictions are located on CSS contigs that have been assigned to POPSEQ cM bins. Gene predictions on contigs which lack such assignment are included in POTAGE to allow convenient access to their functional annotation and expression values even though the utility of those entries is limited. The functional annotations of CSS (ftp://ftpmips.helmholtz-muenchen.de/plants/wheat/IWGSC/genePrediction_v2.1/) were supplemented with rice annotations based on translated sequence similarity. This was done by taking the representative HC transcripts and aligning them to rice coding sequences (RGAP version 7)^11^ using BLASTx^12^ with an e-value cut-off 10^−5^. The functional annotation of the best hit was associated with the HC gene prediction.

The expression values included in POTAGE were computed as described previously^10^ from bread wheat tissue series RNA-Seq data set^2^, which covers five different organs at three developmental stages, each in two replicates. Capturing homoeolog-specific expression in hexaploid wheat based on short reads may be problematic^13^. We have taken great care to achieve that wherever possible. This is subject to homoeologous pairs or triplets being present in the reference and distinct within the sequenced fragment length. Briefly, we generated a reference for aligning the RNA-Seq reads by extracting the genomic sequence for each of the predicted genes with up to 2 kbp upstream and downstream bases whenever available from the corresponding CSS contig. The RNA-Seq reads were quality-, adapter- trimmed using Trimmomatic^14^, version 0.30 and aligned to the reference using TopHat^15^ version 2.2.1, not allowing any mismatches or indels. Paired reads were required to map concordantly to the same reference sequence and the resulting BAM files for the biological replicates were merged. Expression was quantified by Cufflinks^16^ version 2.1.1 utilizing an adjusted version of the reference transcript annotations provided with the HC gene predictions. FPKMs (fragments per kilobase of exon per million fragments mapped) per gene (rather than per isoform) were extracted and aggregated in tabular form.

### Visualisation and interface

**Figure 1:**
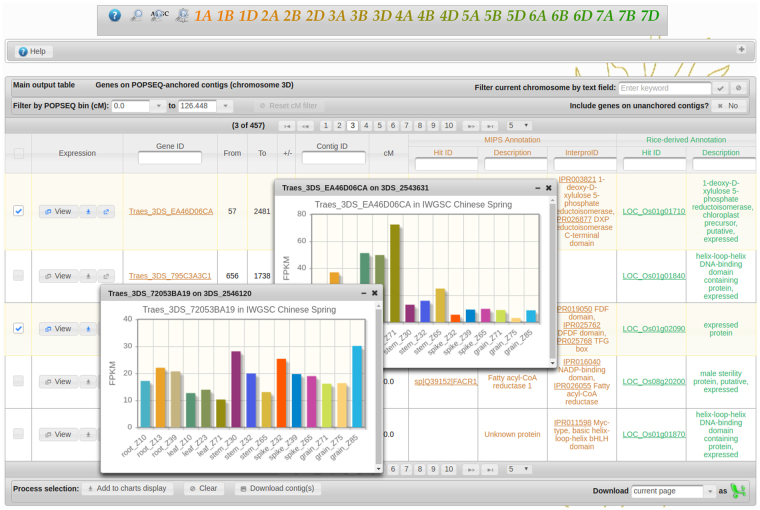
Main view of POTAGE web application, including the navigation dock at the top with the help panel directly underneath. Most of the view is occupied by the main output table, here overlaid with two pop-up dialog components displaying expression charts.

The primary focus in the design of the interface (see Fig. 1) was on the ease with which a user can locate genes of interest. To accommodate this, POTAGE accepts two types of identifiers derived from two of the underlying datasets. These are the CSS contig identifiers and HC gene prediction identifiers, both of which are frequently used by researchers in the field. Such identifiers might correspond to sequences which include, or, are known to be close to, the flanking markers of a QTL. For convenience, the nucleotide sequence of a flanking marker may be used to identify CSS contig containing that sequence. In such case the input sequences are aligned to the CSS using BLASTn^12^ and the alignment results are presented in a way that enables rapid identification of, and navigation to, the relevant positions within POTAGE output tables. The available functional annotation fields provide link-outs to relevant external databases^11,17–20^. Virtually all the annotation fields in POTAGE output table are searchable, allowing the user to filter, and home in on genes of interest. As such, it is not only useful for prioritizing candidate genes under QTL, but can also be used as an entry point to study and aggregate information available for a gene family or a group of genes based on their putative function.

**Figure 2:**
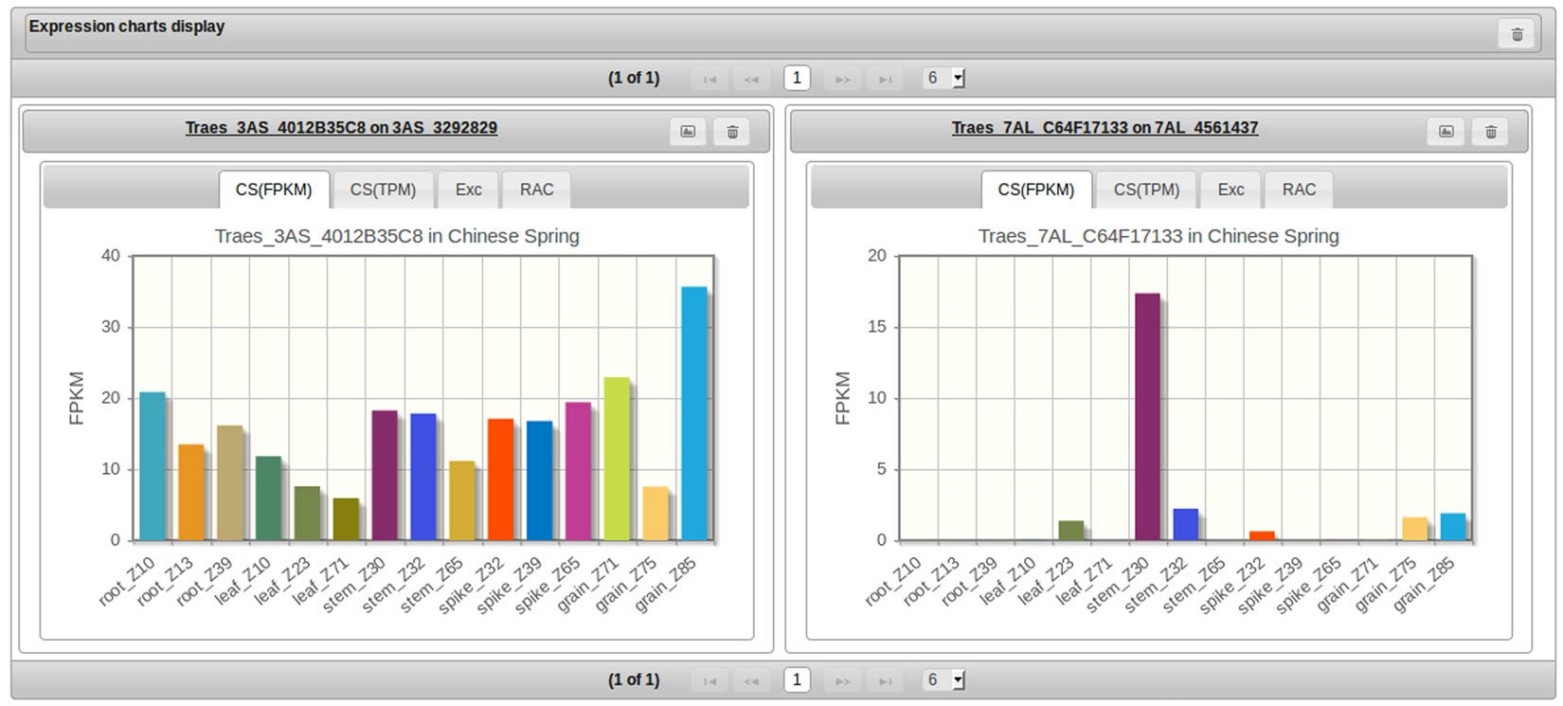
Multiple expression charts can be held by, and, displayed in a panel grid component. This allows the user to retain information about selected genes while navigating through the main output table. Additional tabs over the charts indicate various expression datasets available - these are not included in the public version of POTAGE but additional datasets can be added to a local installation.

The interface provides convenient download/export functionality, giving the user direct access to the data presented in POTAGE. This can be at the level of selected genes, chromosomal regions or entire chromosomes. POTAGE can also list all CSS contigs with POPSEQ assignments, including those without HC genes predictions. These may allow the user to identify the POPSEQ bin/position relevant to a QTL in cases where the input markers cannot be linked with any of the gene predictions displayed in the main output table. The web application is designed in a modular way to allow the display of expression data that may come from multiple RNA-Seq experiments. Expression values are visualised as bar-charts which can be displayed individually (Fig. 1) or collectively in a dedicated panel (Fig. 2) to aid analysis of multiple genes of interest. In addition to visualising the expression, POTAGE provides links allowing the user to explore expression data available for a given gene via WheatExp^8^.

### Implementation

POTAGE was developed primarily in JAVA and JSF utilising PrimeFaces 6.0 component library. It is effectively a single-page, dynamic web application. In the interest of speed and responsiveness the underlying data are precomputed and held in the server’s memory while components such as charts and their containers are generated dynamically. To alleviate any issues caused by the bottleneck of BLASTn sequence alignment, POTAGE is capable of parsing HTTP GET requests, allowing the user to retrieve results of long-running BLAST jobs at a later time or link to specific gene identifiers or chromosomal regions. All source code is available under the Apache 2.0 license via GitHub (https://github.com/CroBiAd/potage). In addition to the public version of POTAGE available at http://crobiad.agwine.adelaide.edu.au/potage, we have also developed a Docker image for quickly deploying POTAGE locally (https://hub.docker.com/r/crobiad/potage/) as well as work-flows showing how to add your own expression data sets to a local installation. This is of particular relevance to those who work with unpublished datasets or would like to deploy POTAGE on their own hardware.

## Candidate gene identification with POTAGE

To illustrate how POTAGE can streamline the process of gene identification we follow on from a recent study focusing on the agronomically important issue of pre-harvest sprouting^21^. Part of that study was concerned with the identification of genes within the *Phs-A1* QTL. Building on earlier work^22^ Shorinola *et al*. identified *wms894* and *xhbe03* as the flanking markers for a locus located on chromosome 4AL. Based on collinearity with a 75 kb region in *Brachypodium distachyon*, they have placed 10 genes on wheat CSS within, or adjacent to, the QTL. The sequence of *wms894* is located in the promoter region of *Traes_4AL_F00707FAF* (OTU-like cysteine protease) and *xhbe03* falls within the 3′ UTR of *Traes_4AL_F99FCB25F* (*AWMP-19-like*)^21^.

By taking the marker sequences, or the above mentioned gene identifiers, we can identify the POPSEQ bin(s) containing the flanking markers. We found a marker in each of two neighbouring bins, one labelled 76.969 cM and the other labelled 77.537 cM as per POPSEQ nomenclature. We next restrict the chromosome 4A view in POTAGE to these bins, for which POTAGE lists 35 predicted genes. Of these, 32 are in the 76.969 cM bin and the remaining 3 genes are in the 77.537 cM bin (see http://crobiad.agwine.adelaide.edu.au/potage?chromosome=4A&cM=76.969–77.537). Eight of the 35 genes have previously been linked to the *Phs-A1* QTL by Shorinola *et al*.^21^. These include 3 which fall just outside the QTL: *Traes_4AL_F00707FAF* (a putative OTU-like cysteine protease family protein) and two *PM-19* genes which were previously reported as candidates for the dormancy QTL^23^. In Shorinola *et al*. however, the *Phs-A1* interval is fine-mapped (in UK germplasm) to 0.3 cM distal to the *PM-19* genes^21^. Four further genes can be excluded from our list based on synteny with *Brachypodium distachyon*, see Table 1, category (V).

**Table 1:**
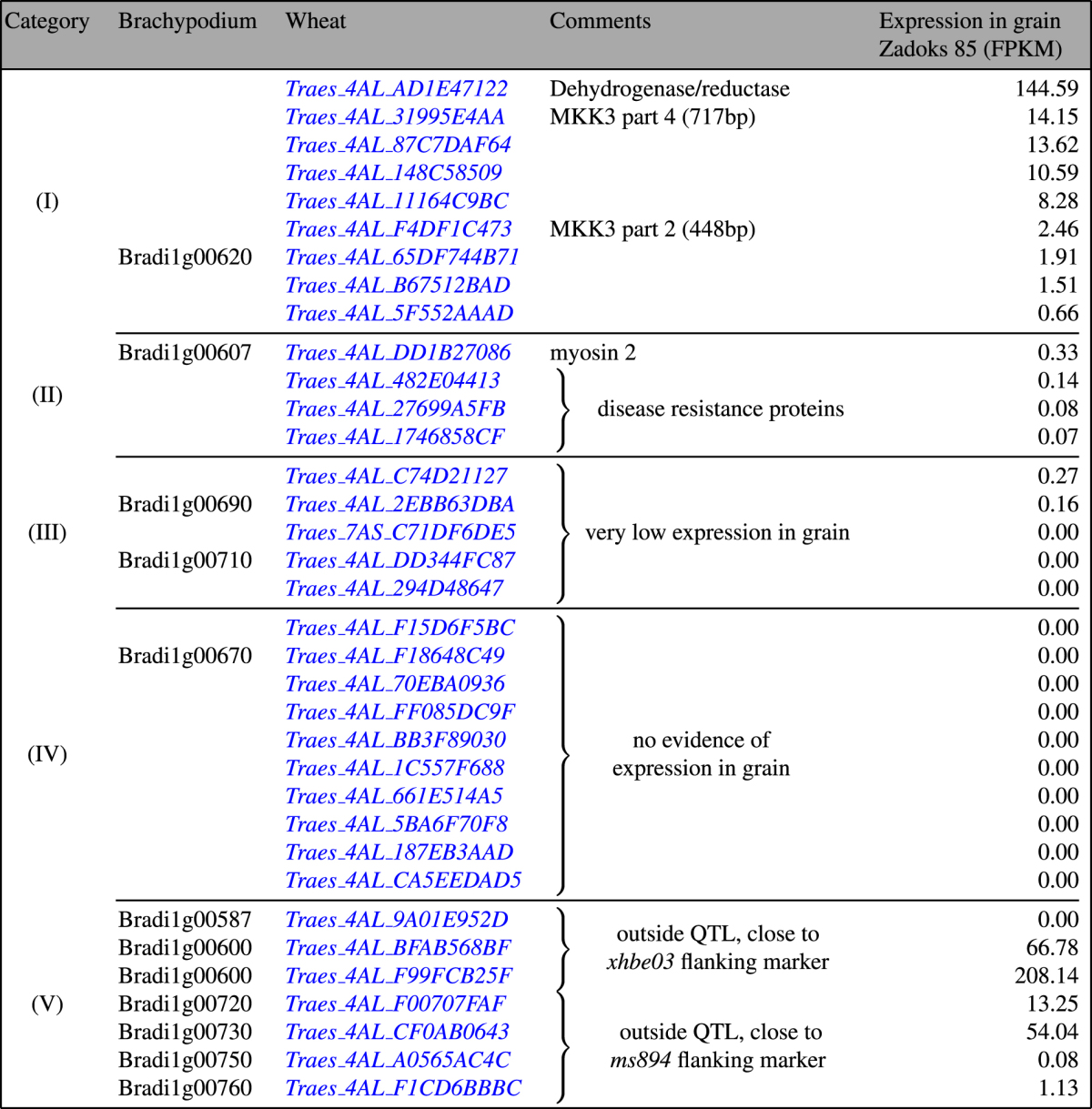
The 35 gene predictions which POTAGE places under and around the *Phs-A1* QTL are distributed between two POPSEQ bins. 32 are in the 76.969 cM bin and the remaining 3 are in the 77.537 cM bin. Categories reflect how the genes were prioritized, with the short list of remaining candidate genes in category (I). The remaining categories group genes depending on the primary reason for their exclusion from the short list of candidates: (II) functional annotation, (III) very low expression in grain, (IV) lack of evidence for expression in grain, (V) map location and synteny to Brachypodium.

Once the user has identified the genes under (or close to) their QTL, and possibly excluded some as described above, it is simple to visualize their expression patterns and to explore their functional annotations in POTAGE. This allows the researcher to prioritise candidate genes depending on the biological context. In the case of the pre-harvest sprouting QTL we analyse the remaining 28 genes to provisionally exclude those with no evidence of expression in grain and focus on those clearly expressed therein. This is done based on the expectation that genes contributing to the trait should be active in grain. We find that 10 genes can provisionally be excluded based on the lack of evidence of those being expressed in grain with further 5 genes treated as weak candidates due to very low expression in grain samples. The functional annotation allows us to tentatively exclude 3 disease resistance genes and a putative myosin gene, which leaves us with 9 short-listed gene-predictions presented in Table 1, category (I).

**Figure 3:**
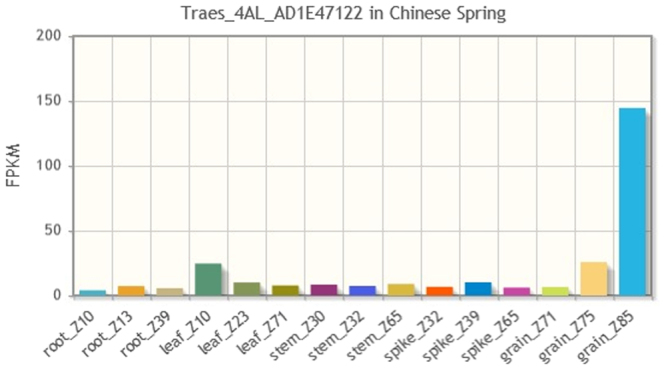
The expression profile of the top candidate, *Traes_4AL_AD1E47122* (annotated as *Dehydrogenase/reductase SDR family protein 7-like*), showing strong expression in grain_Z85.

The gene predictions remaining on our list exhibit at least some evidence of expression in all available tissues and stages. Since we regard grain at Zadoks 85 to be the most relevant for pre-harvest sprouting among the available samples, we next sort our list in descending order of expression in grain_Z85. At the top of the list we now find *Traes_4AL_AD1E47122* annotated as *Dehydrogenase/reductase SDR family protein 7-like*. The expression profile presented in Fig. 3 makes it the top candidate.

**Figure 4:**
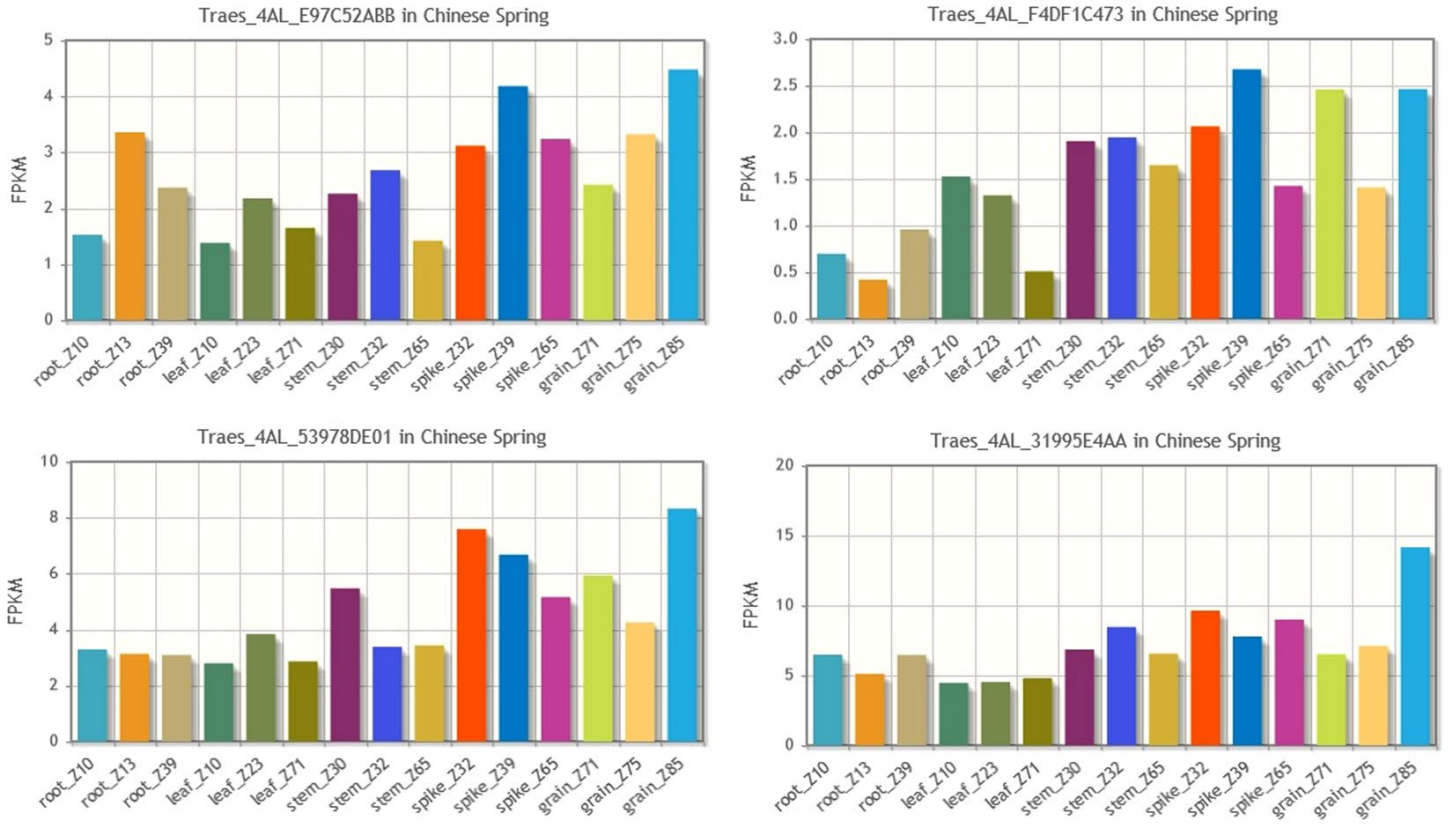
The expression profiles of the four partial gene predictions covering *TaMKK3-A*, charts ordered top-left to bottom-right reflecting how partial predictions cover the gene from 5′ to 3′ end. The transcript lengths for the predictions are: *Traes_4AL_E97C52ABB* 1161 bp, *Traes_4AL_F4DF1C473* 448 bp, *Traes_4AL_53978DE01* 351 bp, *Traes_4AL_31995E4AA* 717 bp.

**Figure 5:**
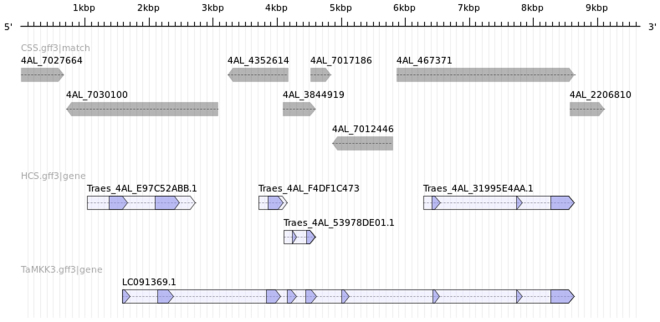
Placement of CSS contigs, the partial HC gene predictions as well the *TaMKK3-A* gene in the coordinate space of *TaMKK3-A* genomic sequence.

Next gene prediction on our list is *Traes_4AL_31995E4AA* annotated as *mitogen-activated protein kinase kinase 3*, we also note that another gene prediction still remaining on our list, *Traes_4AL_F4DF1C473*, carries the same functional annotation–this could indicate e.g. gene duplication, but considering the fragmentation of CSS we first investigate the completeness of the two gene predictions. A BLASTn search against barley sequences confirms that the two gene predictions are indeed partial. Since the two partial predictions do not cover the full length of the gene, we aligned the coding sequence of the barley ortholog to the CSS and identify two further fragments, (*Traes_4AL_E97C52ABB*, *Traes_4AL_53978DE01*) on contigs which lack POPSEQ bin assignments. The expression profiles of the four fragments are presented in Fig. 4. A literature search reveals that Mitogen-activated Protein Kinase Kinase 3 (*TaMKK3-A*), was recently shown to be a causal gene for a major seed dormancy locus in wheat^22^. It is also an ortholog of Barley *MKK3*, which is notable for the N260T mutation which confers an advantage in preventing pre-harvest sprouting^24^. Figure 5 illustrates how the fragmented gene predictions relate to CSS as well the gene and the genomic sequences of *TaMKK3-A* ^22^. The figure was generated using GenomeTools^25^ sketch based on feature descriptions of the HC gene predictions and alignments of CSS and *TaMKK3-A* gene sequence to *TaMKK3-A* genomic sequence using Exonerate^26^. Output of the BLAST-based search in POTAGE led us also to the two putative homeologs of *TaMKK3-A*: *Traes_5DL_F89F21E65* and *Traes_5BL_BB574E77D*. The 5DL prediction is full-length while the 5BL one is fragmented to a lesser extent than its 4AL counterpart. Note that due to a well-known translocation^27,28^ this region of chromosome 4AL corresponds to sections of wheat chromosomes 5B, 5D and barley chromosome 5H, rather than 4B, 4D and 4H respectively.

## Discussion

**Figure 6:**
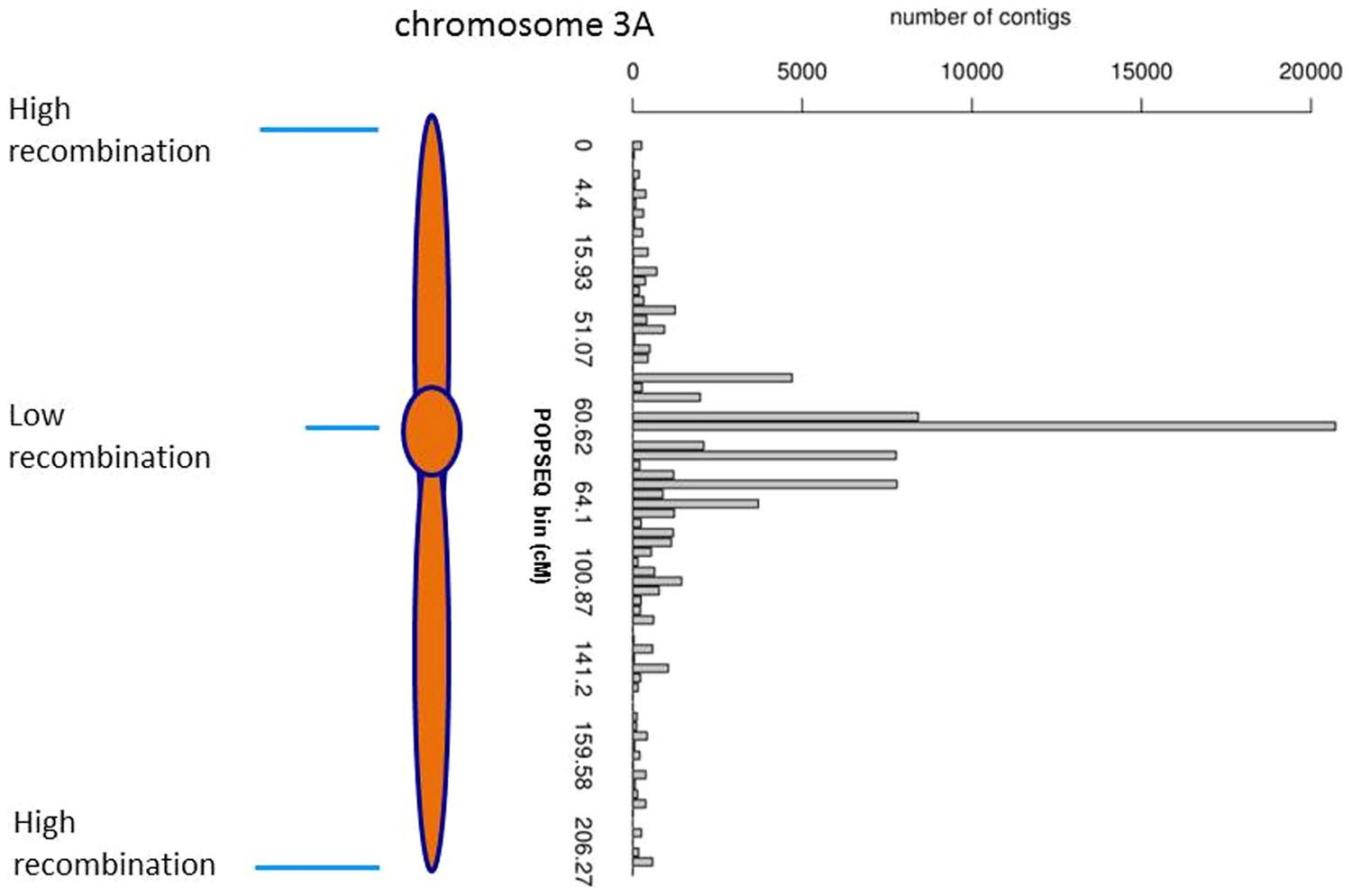
CSS contig frequency in POPSEQ genetic bins along the wheat chromosome 3A illustrates how the resolution of the POPSEQ map is particularly limited around the centromere. POPSEQ bins close to the centromere contain up to tens of thousands of contigs due to suppressed recombination.

Identification of candidate genes under a QTL is often a laborious exercise in gathering and aggregating biologically relevant information from various sources. Access to community-developed data resources coupled with thoughtfully designed software tools such as POTAGE can greatly accelerate the process as illustrated by the example above. For a suitable QTL which is sufficiently distant from the low-recombination region of a chromosome POTAGE is likely to produce a manageable number of candidate genes along with information which allows the user to further narrow-down the search space with the ultimate goal of identifying a causative gene. The user must however be aware of the limitations of this approach, first of which is the low resolution of the POPSEQ map in centromeric regions (Fig. 6) which will lead to identification of large number of candidate genes for QTL in those regions. Another issue stems from the fact that almost 40% of HC gene predictions lack a POPSEQ bin allocation, and such genes will be missed in a typical use case (false-negatives). Furthermore, some of genes listed by POTAGE may fall outside a QTL as relying on POPSEQ information alone we are unable to establish the order of the genes within a given POPSEQ bin (false-positives).

The *Phs-A1* QTL case also highlights other issues, such as the fragmentation of *TaMKK3-A* gene prediction over four separate CSS contigs–this will start to be addressed by the upcoming reference assembly and gene annotations which may be integrated into a future version of POTAGE. Another interesting case among the 35 genes listed for the QTL is *Traes_7AS_C71DF6DE5*. The assignment to chromosome 7AS comes from CSS and is in conflict with the POPSEQ map which places it in the region of this QTL on chromosome 4AL, highlighting inconsistent chromosomal assignments among data sets. In every such case a gene’s location in POTAGE reflects the POPSEQ, rather than CSS, chromosomal assignment, the latter being also available to the user as it constitutes the prefix of a contig identifier. In this particular case, a recent high quality assembly^3^ supports the chromosomal assignment of POPSEQ. Improved, more contiguous assemblies should, in time, result in fewer incorrect assignments. POTAGE also indicates that the two genes listed by Shorinola *et al*. which were absent from the POPSEQ interval for the *Phs-A1* QTL, are present in other POPSEQ bins, *Traes_4AL_02AE47773* was found in the 76.401 cM POPSEQ bin, just outside the region, and *Traes_4AL_C56125840* is reported by POTAGE in the 110.42 cM POPSEQ bin, at a considerable distance to the QTL. Furthermore, *Traes_4AL_C56125840* is the only one of the 10 genes in Shorinola *et al*. which the authors were unable to map within, or link to, the interval due to lack of a genetic marker^21^.

As wheat genome assemblies improve in contiguity over time, we will look to incorporate this new information into POTAGE. In the short term we aim to leverage updated gene predictions such as those recently published^3^. Looking further ahead, we expect new assemblies to contribute pseudomolecule-length representations of individual chromosomes. Because of POTAGE’s gene-centric view, these more contiguous assemblies will improve the precision of results presented by POTAGE and provide the users with a more accurate view of the genes underlying their QTL of interest. With these updates the reliance on POPSEQ for ordering the data will decline over time. However, POPSEQ data will remain essential for genomic regions which have not been assigned to a chromosomal location.

### Data availability

The datasets utilised during the current study are available in the following repositories/locations

- https://urgi.versailles.inra.fr/download/iwgsc/Survey_sequence
- https://dx.doi.org/10.5447/IPK/2014/16
- ftp://ftpmips.helmholtz-muenchen.de/plants/wheat/IWGSC/genePrediction_v2.1
- https://urgi.versailles.inra.fr/files/RNASeqWheat

The code and datasets generated during the current study are available in the following repositories/locations

- https://github.com/CroBiAd/potage
- https://github.com/CroBiAd/potage_data
- https://hub.docker.com/r/crobiad/potage
